# Lipopolysaccharide-enhanced transcellular transport of HIV-1 across the blood-brain barrier is mediated by luminal microvessel IL-6 and GM-CSF

**DOI:** 10.1186/1742-2094-8-167

**Published:** 2011-11-30

**Authors:** Shinya Dohgu, Melissa A Fleegal-DeMotta, William A Banks

**Affiliations:** 1 Department of Pharmaceutical Care and Health Sciences, Faculty of Pharmaceutical Sciences, Fukuoka University, Fukuoka, Japan; 2 Geriatric Research Educational and Clinical Center-St. Louis, St. Louis, MO, USA; 3 Division of Geriatric Medicine, Department of Internal Medicine, Saint Louis University School of Medicine, St. Louis, MO, USA; 4 Biology Department, Clarke University, Dubuque, IA, USA; 5 Geriatric Research Educational and Clinical Center-Veterans Affairs Puget Sound Health Care System, Seattle, WA, USA; 6 Division of Gerontology and Geriatric Medicine, Department of Internal Medicine, University of Washington, Seattle, WA, USA

**Keywords:** Blood-brain barrier, Human immunodeficiency virus type 1, Lipopolysaccharide, Interleukin-6, Granulocyte-macrophage colony-stimulating factor, Mitogen-activated protein kinase

## Abstract

Elevated levels of cytokines/chemokines contribute to increased neuroinvasion of human immunodeficiency virus type 1 (HIV-1). Previous work showed that lipopolysaccharide (LPS), which is present in the plasma of patients with HIV-1, enhanced transcellular transport of HIV-1 across the blood-brain barrier (BBB) through the activation of p38 mitogen-activated protein kinase (MAPK) signaling in brain microvascular endothelial cells (BMECs). Here, we found that LPS (100 μg/mL, 4 hr) selectively increased interleukin (IL)-6 and granulocyte-macrophage colony-stimulating factor (GM-CSF) release from BMECs. The enhancement of HIV-1 transport induced by luminal LPS was neutralized by treatment with luminal, but not with abluminal, antibodies to IL-6 and GM-CSF without affecting paracellular permeability as measured by transendothelial electrical resistance (TEER). Luminal, but not abluminal, IL-6 or GM-CSF also increased HIV-1 transport. U0126 (MAPK kinase (MEK)1/2 inhibitor) and SB203580 (p38 MAPK inhibitor) decreased the LPS-enhanced release of IL-6 and GM-CSF. These results show that p44/42 and p38 MAPK signaling pathways mediate the LPS-enhanced release of IL-6 and GM-CSF. These cytokines, in turn, act at the luminal surface of the BMEC to enhance the transcellular transport of HIV-1 independently of actions on paracellular permeability.

## Background

Human immunodeficiency virus type 1 (HIV-1) infection induces neurological dysfunctions known as the AIDS-dementia complex or HIV-associated dementia (HAD). Although highly active antiretroviral therapy (HAART) and combination antiretroviral therapy (cART) have dramatically decreased the incidence and severity of HAD, the prevalence of HAD, including minor cognitive and motor disorders, is increasing with the longer lifespan of HIV patients [1]. Most antiretroviral drugs comprising HAART have a restricted entry into the brain because of blood-brain barrier (BBB) efflux transporters so that the brain serves as a reservoir for HIV-1 [2] and a source for viral escape [3]. Therefore, HIV-1 in the brain can contribute to the incidence and development of HIV-associated neurological impairment in HIV-1 patients both prior to and after treatment with HAART/cART.

HIV-1 can enter the brain by two routes: the passage of cell-free virus by an adsorptive endocytosis-like mechanism [4-7] and trafficking of HIV-1-infected immune cells across the BBB [8]. HIV-1 infection of brain endothelial cells (BECs) is not a productive infection [9] and penetration of HIV-1 is independent of the CD4 receptor [10]. At the early stage, HIV-1 enters the brain through an intact, normally functioning BBB [11]. At later stages of infection, elevated levels of proinflammatory cytokines/chemokines in the blood of patients with AIDS [12-14] are likely associated with the increase in HIV-1 infiltration [15-17], while HIV-1 gp120 and Tat induce the disruption of tight junctions in BECs [17-20].

As reported by Brenchley et al. and confirmed by others, plasma levels of lipopolysaccharide (LPS), a Gram-negative bacterial endotoxin, are higher in chronic HIV-infected patients with HAART than in the uninfected [3,21]. Bacterial infection in HIV patients influences the severity and rate of disease progression [22]. Peripheral LPS induces various inflammatory and immunological reactions including the production of cytokines/chemokines, such as tumor necrosis factor-α (TNF-αinterleukin (IL)-1, and IL-6 [23-25]. TNF-α enhances HIV-1 transport across the BBB [15] and LPS induces an increase in HIV-1-infected monocyte transport across the BBB [8]. In our previous in vivo study, we found that the peripheral injection of LPS enhanced gp120 uptake by brain [26]. These studies suggest that elevated levels of inflammatory mediators, including cytokines/chemokines and LPS, regulate the permeability of the BBB to HIV-1. BECs express LPS receptors, such as Toll-like receptor (TLR)-2, TLR-4, and CD14 [27] and are targets of LPS. The barrier function of the BBB is affected by various cytokines/chemokines in the blood compartment [28]. Several studies using in vitro BBB models have shown that LPS increases the paracellular permeability of the BBB [29-33]. LPS induces or enhances the secretion of several cytokines by BECs [34]. Thus, bacterial infection and the accompanying inflammatory state could be involved in the enhancement of HIV-1 entry into the brain.

We recently reported that LPS increased transcellular transport of HIV-1 across the BBB through p38 mitogen-activated protein kinase (MAPK) [35]. Here, we examined whether LPS-enhanced release of cytokines by BMECs mediated the transcellular transport of HIV-1 and was regulated by MAPK signaling pathways.

## Materials and methods

### Radioactive labeling

HIV-1 (MN) CL4/CEMX174 (T1) prepared and rendered noninfective by aldrithiol-2 treatment as previously described [36] was a kind gift of the National Cancer Institute, NIH. The virus was radioactively labeled by the chloramine-T method, a method which preserves vial coat glycoprotein activity [37,38]. Two mCi of ^131^I-Na (Perkin Elmer, Boston, MA), 10 μg of chloramine-T (Sigma) and 5.0 μg of the virus were incubated together for 60 sec. The radioactively labeled virus was purified on a column of Sephadex G-10 (Sigma).

### Primary culture of mouse brain microvascular endothelial cells (BMECs)

BMECs were isolated by a modified method of Szabó et al. [39] and Nakagawa et al. [38]. The animals were housed in clean cages in the laboratory with free access to food and water and were maintained on a 12-h dark, 12-h light cycle in a room with controlled temperature (24 ± 1°C) and humidity (55 ± 5%). All procedures involving experimental animals were approved by the local Animal Care and Use Committee and were performed in a facility approved by Association for Assessment and Accreditation of Laboratory Animal Care. Cerebral cortices harvested from 8-week-old male CD-1 mice from our in-house colony were homogenized, BMECs extracted, and cultured as previously performed [40]. Cultures were treated with puromycin to remove pericytes.

### Preparation of in vitro BBB models

BMECs (4 × 10^4 ^cells/well) were seeded on the inside of the fibronectin-collagen IV (0.1 and 0.5 mg/mL, respectively)-coated polyester membrane (0.33 cm^2^, 0.4 μm pore size) of a Transwell^®^-Clear insert (Costar, Corning, NY) placed in the well of a 24-well culture plate (Costar). Culture methods were the same as previously reported [35]. Transendothelial electrical resistance (TEER in Ω × cm^2^) was measured before the experiments and after an exposure of LPS using an EVOM voltohmmeter equipped with STX-2 electrode (World Precision Instruments, Sarasota, FL). The TEER of cell-free Transwell^®^-Clear inserts were subtracted from the obtained values.

### Pretreatment protocol

Lipopolysaccharide from *Salmonella typhimurium *(LPS; Sigma), monoclonal anti-mouse GM-CSF antibody, anti-mouse IL-6 antibody, mouse GM-CSF, and mouse IL-6 (all purchased from R&D systems, Minneapolis, MN) were dissolved in serum-free DMEM/F-12 (DMEM/F-12 containing 1 ng/mL bFGF and 500 nM hydrocortisone). The dose of LPS used in previous BMEC studies (100 μg/mL) was added to the luminal chamber of the Transwell^® ^inserts, and anti-mouse GM-CSF antibody (10 μg/mL), anti-mouse IL-6 antibody (10 μg/mL), mouse GM-CSF (1-100 ng/mL), or mouse IL-6 (1-100 ng/mL) was loaded into the luminal or abluminal chamber. Then, the BMEC monolayers were incubated for 4 hr at 37°C with a humidified atmosphere of 5% CO_2_/95% air. In the experiments using antibodies, rat IgG (Sigma) was added to the control and LPS-treated group (10 μg/mL as final concentration).

U0126 (MEK inhibitor; Tocris Cookson Inc., Ellisville, MO), SB203850 (p38 MAPK inhibitor; Tocris) and SP600125 (Jun kinase (JNK) inhibitor; Sigma) were first dissolved in dimethyl sulfoxide (DMSO) and diluted with serum-free DMEM/F-12 (0.1% as the final DMSO concentration).

### Transendothelial transport of ^131^I-HIV-1

For the transport experiments, the medium was removed and BMECs were washed with physiological buffer containing 1% BSA (141 mM NaCl, 4.0 mM KCl, 2.8 mM CaCl_2_, 1.0 mM MgSO_4_, 1.0 mM NaH_2_PO_4_, 10 mM HEPES, 10 mM D-glucose and 1% BSA, pH 7.4). The physiological buffer containing 1% BSA was added to the outside (abluminal chamber; 0.6 mL) of the Transwell^® ^insert. To initiate the transport experiments, ^131^I-HIV-1 (3 × 10^6 ^cpm/mL) was loaded on the luminal chamber. The side opposite to that to which the radioactive materials were loaded is the collecting chamber. Samples (0.5 ml) were removed from the abluminal chamber at 15, 30, 60 and 90 min and immediately replaced with an equal volume of fresh 1% BSA/physiological buffer. All samples were mixed with 30% trichloroacetic acid (TCA; final concentration 15%) and centrifuged at 5, 400 ×g for 15 min at 4°C. Radioactivity in the TCA precipitate was determined in a gamma counter. The permeability coefficient and clearance of TCA-precipitable 131I-HIV-1 was calculated according to the method described by Dehouck et al. [41]. Clearance was expressed as microliters (μL) of radioactive tracer diffusing from the luminal to abluminal (influx) chamber and was calculated from the initial level of radioactivity in the loading chamber and final level of radioactivity in the collecting chamber:

$$\text{Clearance}\left(\mu \text{L}\right)={\left[\text{C}\right]}_{\text{C}}\times V_{\text{C}}∕{\left[\text{C}\right]}_{\text{L}},$$

where [C]_L _is the initial radioactivity in a microliter of loading chamber (in cpm/μL), [C]_C _is the radioactivity in a microliter of collecting chamber (in cpm/μL), and *V*_C _is the volume of collecting chamber (in μL). During a 90-min period of the experiment, the clearance volume increased linearly with time. The volume cleared was plotted versus time, and the slope was estimated by linear regression analysis. The slope of clearance curves for the BMEC monolayer plus Transwell^® ^membrane was denoted by *PS*_app_, where *PS *is the permeability × surface area product (in μL/min). The slope of the clearance curve with a Transwell^® ^membrane without BMECs was denoted by *PS*_membrane_. The real *PS *value for the BMEC monolayer (*PS*_e_) was calculated from

$$1∕PS_{\text{app}}=1∕PS_{\text{membrane}}+1∕PS_{\text{e}}.$$

The *PS*_e _values were divided by the surface area of the Transwell^® ^inserts (0.33 cm^2^) to generate the endothelial permeability coefficient (*P*_e_, in cm/min).

### Cytokine detection

BMECs (4 × 10^4 ^cells/well) were seeded on the fibronectin/collagen I/collagen IV (0.05, 0.05, and 0.1 mg/mL, respectively)-coated 24-well culture plate (Costar). BMECs were washed with serum-free DMEM/F-12, and then exposed to 200 μL of LPS (100 μg/mL) with or without U0126 (10 μM), SB203580 (10 μM), and SP600125 (10 μM) for 4 hr at 37°C. Culture supernatant was collected and stored at -80°C until use. The cytokines (GM-CSF, IFN-γ, IL-1α, IL-1β, IL-2, IL-4, IL-6, IL-10, IL-12 (p70), and TNF-α) were measured with the mouse cytokine/chemokine Lincoplex^® ^kit (Linco Research, St. Charles, MO) by following the manufacturer's instructions.

### Western blot analysis

LPS, GM-CSF, or IL-6-treated and control BMECs were washed three times with ice-cold phosphate buffered saline containing 1 mM sodium orthovanadate (Na_3_VO_4_) and 1 mM sodium fluoride (NaF). Cells were scraped and lysed in phosphoprotein lysis buffer (10 mM Tris-HCl, pH 6.8, 100 mM NaCl, 1 mM EDTA, 1 mM EGTA, 10% glycerol, 1% Triton X-100, 0.1% SDS, 0.5% sodium deoxycholate, 20 mM sodium pyrophosphate decahydrate, 2 mM Na_3_VO_4_, 1 mM NaF, 1 mM phenylmethylsulfonyl fluoride) containing 1% protease inhibitor cocktail (Sigma) on ice. Cell lysates were centrifuged (15, 000 ×g at 4°C for 15 min) and the supernatants were stored at -80°C until use. The protein concentration of each sample was determined using a BCA protein assay kit (Pierce, Rockford, IL). Twenty to thirty μg of the total protein was mixed with NuPAGE^® ^LDS sample buffer (Invitrogen) and incubated for 3 min at 100°C. Proteins were separated on NuPAGE^® ^Novex 4-12% Bis-Tris gel (Invitrogen) and then transferred to a polyvinylidene difluoride (PVDF) membrane (Invitrogen). After transfer, the blots were blocked with 5% BSA/Tris-buffered saline (TBS: 20 mM Tris-HCl, pH 7.5, 150 mM NaCl) containing 0.05% Tween 20 (TBS-T) for 1 hr at room temperature. The membrane was incubated with the primary antibody diluted in 5% BSA/TBS-T overnight at 4°C. The phosphorylation of p44/42 MAPK, p38 MAPK and JNK were detected using anti-phospho-p44/42 MAPK (1:1000), anti-phospho-p38 MAPK (1:500) and anti-phospho-JNK (1:500) rabbit monoclonal antibodies, respectively (all purchased from Cell Signaling Technology, Beverly, MA). Occludin, claudin-5, and ZO-1 were detected using anti-occludin, anti-claudin-5, and anti-ZO-1 mouse monoclonal antibodies (all purchased from Zymed, South San Francisco, CA). Blots were washed and incubated with horseradish peroxidase-conjugated anti-mouse IgG or anti-rabbit IgG (1:10, 000; Santa Cruz Biotechnology, Santa Cruz, CA) diluted in 5% BSA/TBS-T for 1 hr at room temperature. The immunoreactive bands were visualized on an X-ray film (Kodak) using SuperSignal^® ^West Pico chemiluminescent substrate kit (Pierce). To reprobe total p44/42 MAPK, p38 MAPK, JNK, and actin, the membrane was incubated in stripping buffer (0.2 M glycine, 0.1% SDS and 1% Tween 20, pH 2.2) for 15 min twice and blocked with 5% non-fat dry milk/TBS-T. The total p44/42 MAPK, p38 MAPK and JNK were detected using anti-p44/42 MAPK (1:1000), p38 MAPK (1:1000), JNK (1:1000) (all purchased from Cell Signaling Technology), and actin (1:1000; Santa Cruz Biotechnology) antibodies, respectively. To quantify the relative levels of protein expression, the intensity of specific protein bands was quantified using ImageJ software (National Institute of Health, Bethesda, MD) and then normalized by that of each loading control protein.

### Statistical analysis

Values are expressed as means ± SEM. One-way and two-way analysis of variances (ANOVAs) followed by Dunnett's or Tukey-Kramer's test were applied to multiple comparisons. Paired t-test was applied to the densitometry analysis. The differences between means were considered to be significant when *P *values were less than 0.05 using Prism 5.0 (GraphPad, San Diego, CA).

## Results

### LPS stimulated release of GM-CSF and IL-6 by BMEC

As shown in Table 1, BMECs spontaneously secreted IL-1β, IL-2, IL-4, IL-10, IL-12, and TNF-α in the 0.5-2.5 pg/mL range, and GM-CSF, IFN-γ, and IL-6 in 4-7 pg/mL range in this study. The concentration of IL-1α was below the detection level of the assay. A 4 hr exposure of BMECs to LPS (100 μg/mL) significantly induced 33- and 2.4-fold increases in the levels of GM-CSF and IL-6, respectively (*P *< 0.01). LPS significantly decreased the secretion of IFN-γ by BMECs (*P *< 0.01), but the decrease in the secretion of IL-12 with LPS did not reach statistical significance. Secretion of IL-1β, IL-2, and IL-10 was not detected after LPS treatment. The level of IL-4 and TNF-α did not change after LPS treatment.

**Table 1:** Effect of LPS on the release of cytokines by BMECs.

	Treatment	
	
Cytokine (pg/mL)	Control	LPS 100 μg/mL
GM-CSF	4.8 ± 3.1	160.0 ± 21.7**
IFN-γ	4.4 ± 1.1	1.6 ± 0.6*
IL-1α	N.D.	N.D.
IL-1β	N.D.	N.D.
IL-2	1.1 ± 0.5	N.D.
IL-4	0.9 ± 0.2	0.5 ± 0.2
IL-6	6.7 ± 1.1	16.3 ± 2.3**
IL-10	2.3 ± 1.1	N.D.
IL-12 (p70)	1.6 ± 0.6	0.3 ± 0.3
TNF-α	0.5 ± 0.3	0.2 ± 0.2

BMECs were exposed to LPS (100 μg/mL) for 4 hr. Mean ± SEM (n = 7). N.D.: Not Detected. Statistical analysis was done by Student's t-test. * *P *< 0.05, ** *P *< 0.01 vs control.

### Polarized effect of antibodies to IL-6 and GM-CSF on LPS-induced increase in HIV-1 permeability and paracellular permeability of BMEC monolayer

**Figure 1:**
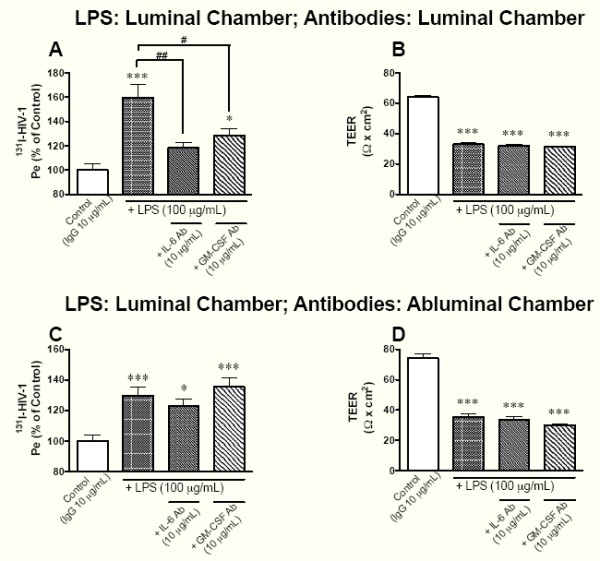
**Effects of antibodies to IL-6 and GM-CSF on LPS-induced changes in the permeability of BMECs to ^131^I-HIV-1 (A and C) and TEER (B and D)**. LPS (100 μg/mL) was added to the luminal chamber. In panels A and B, antibodies to IL-6 or GM-CSF were added to the luminal chamber. In panels C and D, antibodies to IL-6 or GM-CSF were added to the abluminal chamber. After 4 hr of incubation, transport study was performed. In panels A and C, results are expressed as % of control. Control values were 1.52 ± 0.16 × 10^-5 ^and 1.52 ± 0.05 × 10^-5 ^cm/min (A and C, respectively). Values are means ± SEM (n = 9-15). *P < 0.05, ***P < 0.001, significant differences from control. ^#^*P *< 0.05, ^##^*P *< 0.01, significant differences from LPS (100 μg/mL).

To examine whether the enhanced release of IL-6 and GM-CSF induced by LPS was involved in the LPS-induced increases in HIV-1 permeability and paracellular permeability of the BMEC monolayer, we exposed BMEC monolayers to LPS with antibodies to IL-6 and GM-CSF. Since BMECs can release cytokines from either their luminal or abluminal surface [34], we examined the functional polarity of antibodies to IL-6 and GM-CSF by adding them into the luminal or abluminal chambers. We assessed the paracellular permeability of the BMEC monolayer by measuring TEER. LPS (100 μg/mL for 4 hr) added to the luminal chamber significantly increased ^131^I-HIV-1 permeability of BMEC monolayers (Figure 1A and 1C) and decreased TEER (Figure 1B and 1D). The presence of antibodies to IL-6 and GM-CSF (10 μg/mL, respectively) in the luminal chamber significantly attenuated the LPS-induced increase in ^131^I-HIV-1 (Figure 1A), but not the LPS-induced decrease in TEER (Figure 1B). In contrast, antibodies added into the abluminal chamber did not inhibit the LPS-induced increase in ^131^I-HIV-1 permeability and the decrease in TEER (Figure 1C and 1D).

### Polarized response to IL-6 and GM-CSF in the permeability of BMEC monolayer

**Figure 2:**
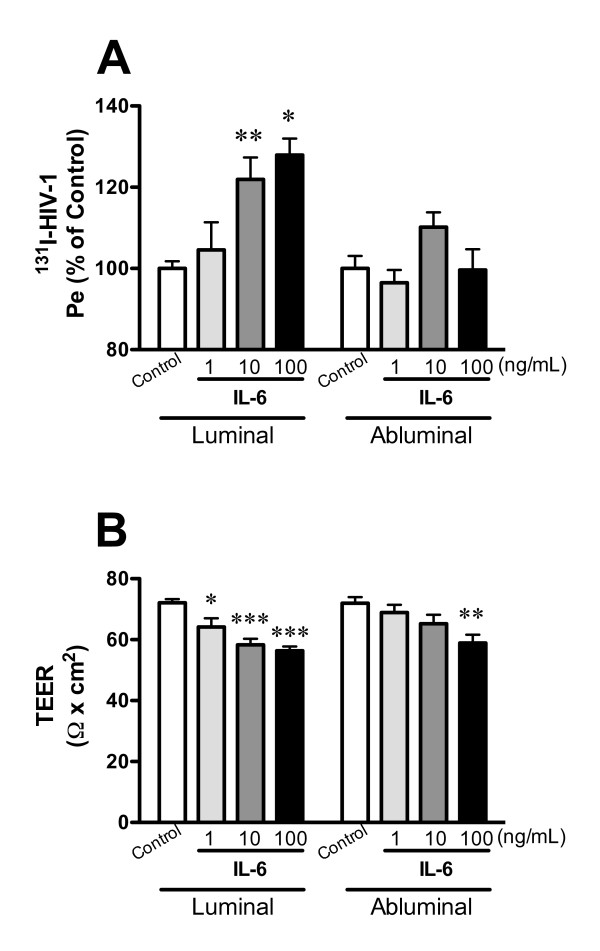
**Functional polarity to IL-6 in BMEC permeability of HIV-1 (A) and TEER (B)**. BMECs were exposed to IL-6 (1, 10, and 100 ng/mL) in the luminal or abluminal chamber for 4 hr. In panel A, results are expressed as % of control. The control values of permeability coefficient for ^131^I-HIV-1 in panel A was 1.03 ± 0.11 × 10^-5 ^and 1.07 ± 0.08 × 10^-5 ^cm/min for the luminal and abluminal control, respectively. Values are means ± SEM (n = 3-12). *P < 0.05, **P < 0.01, ***P < 0.001, significant difference from each corresponding control.

To determine whether IL-6 and GM-CSF mediate HIV-1 transport across the BBB and decrease TEER with the functional polarity, BMECs were treated with various concentrations of mouse IL-6 and GM-CSF (1-100 ng/mL, respectively) in the luminal or abluminal chamber. In Figure 2A, luminal treatment with IL-6 (1, 10, and 100 ng/mL) increased HIV-1 transport to 104.6 ± 6.8, 121.9 ± 5.4, and 127.9 ± 4.1% of control, but abluminal treatment did not induce significant changes in HIV-1 transport (96.5 ± 3.2, 110.2 ± 3.6, and 99.6 ± 5.0% of control). Luminal treatment with IL-6 (1, 10, and 100 ng/mL) significantly decreased TEER (Figure 2B) from 72.1 ± 1.2 to 64.2 ± 2.8, 58.3 ± 2.0, and 56.4 ± 1.4 Ω × cm^2^. Abluminal treatment with IL-6 significantly decreased TEER from 72.0 ± 2.0 to 58.9 ± 2.7 Ω × cm^2 ^at the concentration of 100 ng/mL. For the permeability to HIV-1 (Figure 2A), a two-way ANOVA showed significant effects for the factors "loading chamber" (luminal or abluminal) [*F*(1, 67) = 11.42, *P *< 0.01], concentration [*F*(3, 67) = 5.715, *P *< 0.01], and interaction (loading chamber × concentration) [*F*(3, 67) = 2.788, *P *< 0.05]. For TEER (Figure 2B), a two-way ANOVA showed a significant effect for concentration [*F*(3, 58) = 10.08, *P *< 0.001], but not for loading chamber and interaction.

**Figure 3:**
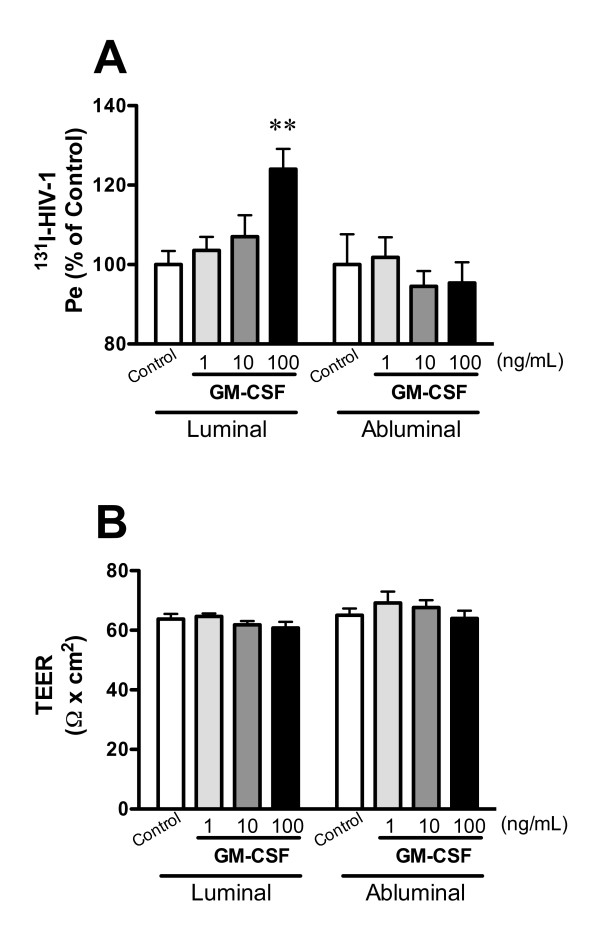
**Functional polarity to GM-CSF in BMEC permeability of HIV-1 (A) and TEER (B)**. BMECs were exposed to GM-CSF (1, 10, and 100 ng/mL) in the luminal or abluminal chamber for 4 hr. In panel A, results are expressed as % of control. The control values of permeability coefficient for ^131^I-HIV-1 in panel A was 137 ± 0.13 × 10^-5 ^and 1.32 ± 0.13 × 10^-5 ^cm/min for the luminal and abluminal control, respectively. Values are means ± SEM (n = 3-12). **P < 0.01, significant difference from control.

As shown in Figure 3A, GM-CSF (1, 10, 100 ng/mL) in the luminal chamber increased HIV-1 transport to 103.6 ± 3.4, 107.0 ± 5.4, and 124.0 ± 5.1% of control, but GM-CSF in the abluminal chamber did not (101.8 ± 5.1, 94.5 ± 3.9, and 95.4 ± 5.2% of control). Neither the luminal nor abluminal treatments with GM-CSF changed TEER (Figure 3B). For the permeability to HIV-1 (Figure 3A), a two-way ANOVA showed significant effects for loading chamber [*F*(1, 44) = 7.746, *P *< 0.01] and interaction [*F*(3, 44) = 2.909, *P *< 0.01] but not concentration. For TEER (Figure 3B), a two-way ANOVA showed a significant effect for loading chamber [*F*(1, 74) = 4.682, *P *< 0.05] but not concentration or interaction.

These results indicated that the effects of LPS on BMECs permeability to HIV-1 were mainly mediated by IL-6 and GM-CSF acting at the luminal surface of the BMEC. In all subsequent studies, therefore, we employed the luminal chamber as the loading chamber.

### Effects of LPS, IL-6, and GM-CSF on the expression of tight junction proteins

**Figure 4:**
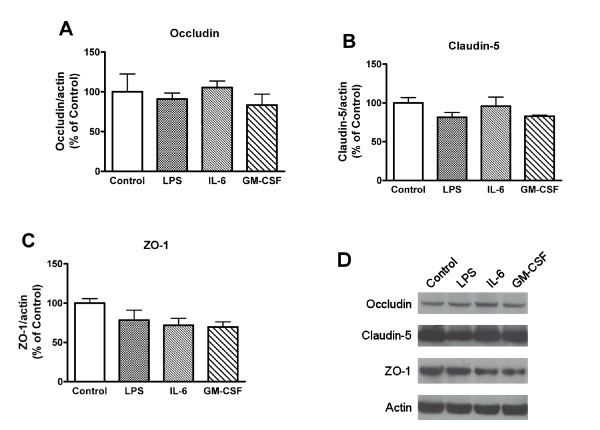
**Effects of LPS, IL-6, and GM-CSF on the expression of tight junction proteins in BMECs**. BMECs were exposed to LPS (100 μg/mL), IL-6 (100 ng/mL), or GM-CSF (100 ng/mL) for 4 hr. Expression levels of occludin, claudin-5, ZO-1, and actin were detected by western blot. Relative intensity of occludin (A), claudin-5 (B), and ZO-1 (C) is calculated as ratio of arbitrary densitometric units of target protein to that of actin. Results are expressed % of control. Values are means ± SEM (n = 3). (D) Photographs are representative in three independent experiments.

To examine the effects of LPS, IL-6, and GM-CSF on the expression of tight junction proteins, BMECs were exposed to LPS (100 μg/mL), IL-6 (100 ng/mL), and GM-CSF (100 ng/mL) for 4 hr (Figure 4). The densitometry analysis showed that there were no significant changes in the expression of tight junction proteins induced by LPS, IL-6, and GM-CSF.

### Effect of MAPK inhibitors on the release of IL-6 and GM-CSF enhanced by LPS

**Figure 5:**
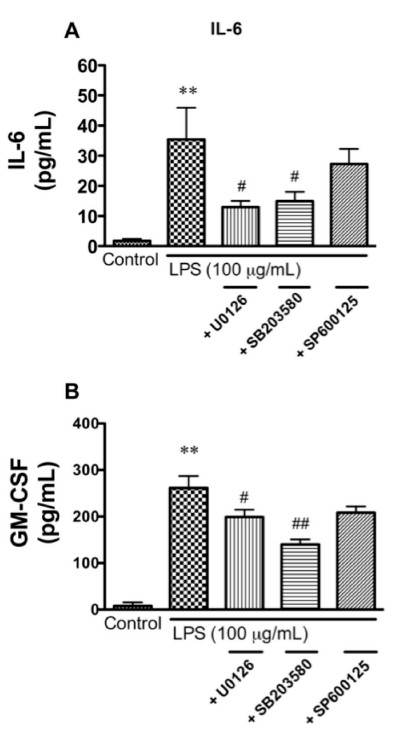
**Effects of various MAPK inhibitors on LPS-enhanced release of IL-6 (A) and GM-CSF (B) by BMECs**. BMECs were treated with LPS (100 μg/mL) for 4 hr in the presence or absence of U0126 (10 μM), SB203580 (10 μM), or SP600125 (10 μM). Values are means ± SEM (n = 5-8). **P < 0.01, significant differences from control. ^#^*P *< 0.05, ^##^*P *< 0.01, significant differences from LPS (100 μg/mL).

We previously demonstrated that LPS activated p44/42 MAPK and p38 MAPK pathways in BMECs [35]. To test whether LPS enhances the release of IL-6 and GM-CSF by BMECs through MAPK signaling pathways, BMECs were exposed to LPS with various MAPK inhibitors (U0126, SB203580, and SP600125) for 4 hr. As shown in Figure 5A and 5B, LPS significantly enhanced the release of IL-6 and GM-CSF by BMECs from 1.7 ± 0.71 to 35.5 ± 10.5 pg/mL (*P *< 0.01) and from 7.8 ± 7.8 to 261.4 ± 25.7 pg/mL (*P *< 0.001), respectively. In the presence of 10 μM of U0126 (MEK1/2 inhibitor), the LPS-induced increase in the release of IL-6 and GM-CSF by BMECs was significantly decreased to 13.0 ± 2.1 (*P *< 0.05 vs LPS) and 199.0 ± 16.0 pg/mL (*P *< 0.05 vs LPS), respectively. Similarly, SB203580 (10 μM: p38 MAPK inhibitor) significantly decreased the LPS-enhanced release of IL-6 and GM-CSF by BMECs to 14.9 ± 3.1 (*P *< 0.05 vs LPS) and 139.9 ± 10.8 pg/mL (*P *< 0.01 vs LPS). The JNK inhibitor SP600125 (10 μM) did not affect the LPS-enhanced release of IL-6 and GM-CSF.

### Effects of IL-6 and GM-CSF on phosphorylation of p44/42 MAPK, p38, and JNK

**Figure 6:**
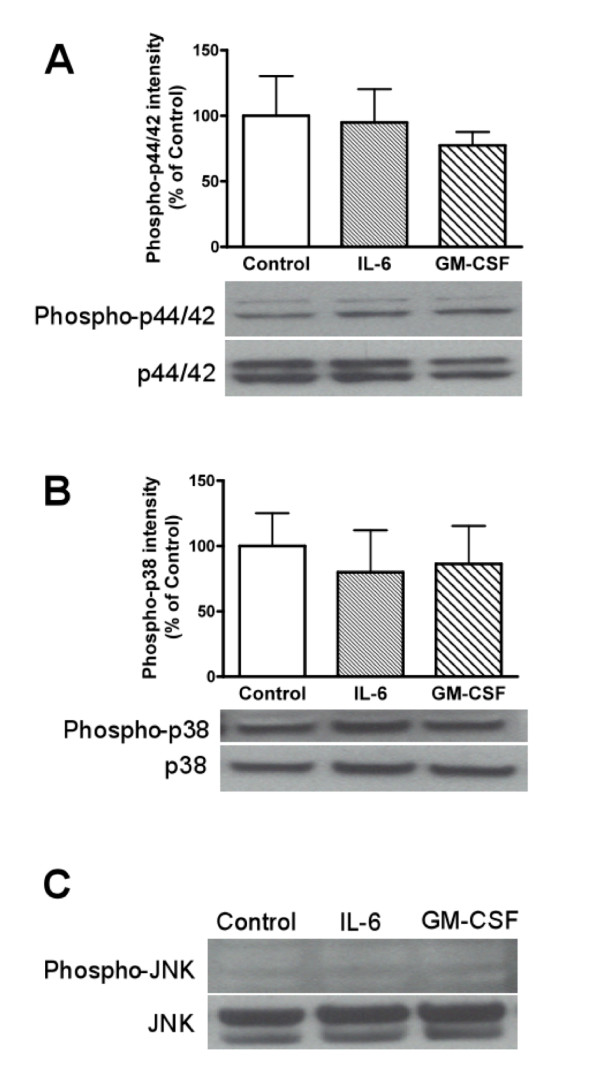
**Effects of IL-6 and GM-CSF on phosphorylation of MAPKs in BMECs**. BMECs were exposed to IL-6 (100 ng/mL) or GM-CSF (100 ng/mL) for 4 hr. Western blot analyses were performed to detect phosphorylated p44/42 MAPK (A), p38 MAPK (B), and JNK (C) as well as total p44/42 MAPK, p38 MAPK, and JNK. Relative intensity is calculated as ratio of arbitrary densitometric units of phoshorylated protein to that of total protein. (C) For phospho-JNK, sorbitol-treated PC12 cells were used as positive control (data not shown). Results are expressed % of control. Values are means ± SEM (n = 3-4). Photographs are representative in three to four independent experiments.

To determine whether IL-6 and GM-CSF could activate MAPK pathways in BMECs as in the case of LPS phosphorylation of MAPKs were measured by western blot analysis (Figure 6). A 4 hr exposure of BMECs to IL-6 (100 ng/mL) or GM-CSF (100 ng/mL) in the luminal side did not increase the phosphorylation of p44/42 MAPK, p38, or JNK.

## Discussion

**Figure 7:**
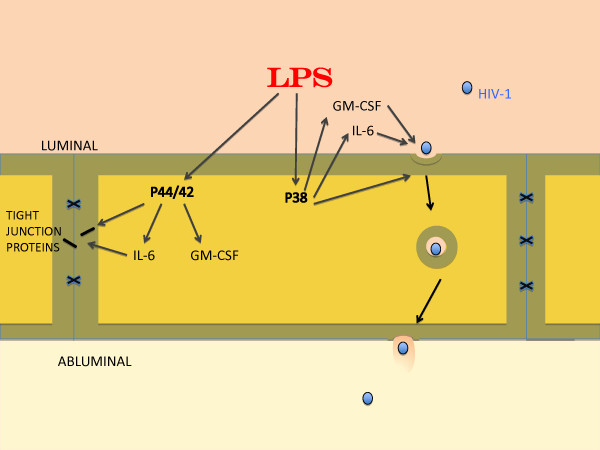
**Schematic of findings.** LPS released both GM-CSF and IL-6 through MAPKs p44/42 and p38 pathways. Previous work has shown that LPS-induced tight junction disruption (paracellular permeability) is mediated through the p44/42 MAPK pathway whereas HIV-1 transcytosis is mediated through the p38 MAPK pathway. Tight junction function as measured by TEER but not tight junction protein expression as measured by western blots was influenced by IL-6 (but not GM-CSF) through a site not blocked by antibodies and so assumed to be intracellular. GM-CSF and IL-6 both promoted HIV-1 transcytosis. The transcytotic effects of GM-CSF and IL-6 were mediated through luminal but not abluminal sites that were blocked by antibodies and therefore assumed to be extracellular.

The present study evaluated whether the LPS-enhanced transcellular transport of HIV-1 across BMEC monolayers was mediated through the induction of the release of cytokines from BMECs. Our main findings are summarized in Figure 7. BMECs spontaneously secreted GM-CSF, IFN-γ, IL-2, IL-4, IL-6, and TNF-α (Table 1) with relatively high concentrations (> 4 pg/mL) of IL-6, GM-CSF, and IFN-γ. LPS markedly increased the concentrations of IL-6 and GM-CSF. Therefore, we hypothesized that IL-6 and/or GM-CSF might mediate the LPS-induced increase in HIV-1 transport across the BBB. Previously, we showed that BMECs in which pericytes were not removed spontaneously secrete GM-CSF, IL-1α, IL-6, IL-10, and TNF-α and that LPS stimulates the secretion of GM-CSF, IL-6, IL-10, and TNF-α [34]. In the current study, the LPS-induced increase in IL-10 and TNF-α secretion was not observed. This may be attributed to the differences of culture conditions, such as the use of culture medium containing hydrocortisone, absence of pericytes, or differences among batches of LPS. Although hydrocortisone inhibits the production of TNF-α by LPS-stimulated monocytes [42], the concentration of hydrocortisone that we used was at a physiological level [43].

BBB disruption can occur either [44] through the paracellular route (increased leakage between cells as measured by a decrease in TEER) or though the transcellular route (increased passage across a cell). Viral-sized particles [45], including HIV-1 [7], generally cross by the transcellular route. Our previous work found that LPS both increased the transcellular permeability of the BMEC monolayer to HIV-1 and decreased TEER [35]. Here, we examined whether IL-6 and GM-CSF release from BMEC by LPS mediated these effects. The presence of LPS and antibodies to IL-6 or GM-CSF in the luminal chamber attenuated LPS-enhanced HIV-1 transport across the BMEC monolayer without a change in TEER (Figure 1A and 1B). BMECs secrete IL-6 and GM-CSF into both the luminal and abluminal chambers [34]. To determine whether IL-6 and GM-CSF secreted by BMECs into the abluminal chamber are also involved in the LPS-induced increase in HIV-1 transport, we added antibodies to IL-6 or GM-CSF to the abluminal chamber. Neither antibody in the abluminal chamber inhibited the luminal LPS-induced changes in HIV-1 transport and TEER (Figure 1C and 1D). These results show that the IL-6 and GM-CSF secreted by BMECs in response to luminal exposure to LPS act at the luminal, but not the abluminal, endothelial surface to increase the transcellular permeability of BMECs to HIV-1. Furthermore, the results suggest that the LPS-induced increase in the paracellular permeability of the BMEC monolayer as measured by TEER is not mediated by extracellular IL-6 and GM-CSF.

We further investigated this functional polarity by adding IL-6 and GM-CSF to the luminal or abluminal chamber. Polarity of other cytokine actions has been investigated. We previously found that BMECs show no functional polarity in the reduction of paracellular permeability by transforming growth factor (TGF)-β1 [46]. That is, either luminal or abluminal TGF-β1 has the same effect on the BBB paracellular permeability. In contrast, MCP-1 is only able to stimulate monocyte migration across BMECs when added to the abluminal surface [47]. In the current study, only luminal IL-6 increased HIV-1 transport and was 10-100 fold more potent than abluminal IL-6 in decreasing TEER (Figure 2). Consistent with this, de Vries et al. reported that IL-6 increased paracellular permeability of BMECs [48]. However, we found here that the IL-6-induced decrease in TEER was less than the LPS-induced decrease in TEER. Other soluble factors, such as other cytokines or chemokines, may be responsible for the remaining increase in the paracellular permeability induced by LPS. An IL-6-independent, P44/42-mediated phosphorylation of tight junction proteins may also be operational. The ability of IL-6 to decrease TEER but an inability of IL-6 antibody to block the effect of LPS on TEER suggests either that the LPS effect is not mediated through IL-6 or that IL-6 acts at a site not available to antibodies, such as inside the cell. Abluminal IL-6 (100 ng/mL) did not alter HIV-1 permeability despite the decrease in TEER. This finding is consistent with IL-6 promoting a transcellular or transcytotic mechanism for HIV-1 passage across the BBB that is independent of the paracellular pathway.

Luminal GM-CSF at the concentration of 100 ng/mL increased HIV-1 transport, whereas abluminal GM-CSF did not. Neither luminal nor abluminal GM-CSF changed TEER (Figure 3). This result further supports the idea that HIV-1 penetration across the BBB is through the transcellular route rather than the paracellular route. In addition, these results may suggest that the receptors for IL-6 and GM-CSF that affect HIV-1 permeability are mainly localized to the luminal membrane of BMECs. Therefore, enhanced invasion of HIV-1 into the brain may be mediated by BMEC-derived cytokines secreted into blood or by blood-borne cytokines. Consistent with this, IL-6 in the blood compartment induces BBB dysfunction [48,49]. As summarized above, LPS, IL-6, and GM-CSF altered both HIV-1 permeability and TEER. The disparities discussed above between these two parameters of BBB function make it likely that they are separate events. Whereas the increased permeability to HIV-1 is likely mediated through transcytotic mechanisms, the decrease in TEER is caused by increased paracellular permeability resulting from altered tight junction function. LPS is known to alter the intensity and pattern of immunohistochemistry for the tight junction proteins claudin-5, ZO-1, and F-actin in BMECs [31,33]. We examined whether LPS, IL-6, and GM-CSF affected the expression of these tight junction proteins in our models (Figure 4). The luminal treatment with LPS, IL-6, or GM-CSF did not induce significant changes in the expression of tight junction proteins in BMECs. Therefore, under the conditions of our model, LPS and IL-6 are likely increasing paracellular permeability of BMECs by altering tight junction function rather than expression of their proteins. For example, LPS and IL-6 may affect the localization of tight junction proteins in BMECs to increase the paracellular permeability.

Our previous work showed that LPS activated p44/42 MAPK and p38 MAPK in BMECs, and the activation of p38 MAPK resulted in the increase in HIV-1 transport [35]. The activation of the p38 MAPK pathway leads to the production and release of inflammatory cytokines [50]. Considering our present results, we hypothesized that either (i) LPS induced the production of IL-6 and GM-CSF through MAPKs or (ii) IL-6 and GM-CSF activated MAPKs. First, we determined whether the LPS-enhanced release of IL-6 and GM-CSF was mediated by MAPK signaling pathways as shown by the experiments using U0126 (MEK1/2 inhibitor), SB203580 (p38 MAPK inhibitor), and SP600125 (JNK inhibitor) (Figure 5). U0126 and SB203580 inhibited the LPS-enhanced release of IL-6 and GM-CSF by BMECs. In the SP600125-treated group, inhibitory effects were not detected. This is reasonable as an LPS-induced increase in the phosphorylation of JNK has not been detected [35]. These results indicated that LPS enhanced the release of IL-6 and GM-CSF from BMECs through the phosphorylation of p44/42 MAPK and p38 MAPK. Thus, the transcellular pathway taken by free virus differs from the JNK dependent, CD40-mediated pathway used by infected monocytes to cross the BBB [3].

Next, we determined whether IL-6 and GM-CSF increased the phosphorylation of MAPKs. IL-6 and GM-CSF did not increase the phosphorylation of p44/42 MAPK, p38 MAPK, or JNK (Figure 6). These results indicated that the IL-6- and GM-CSF-induced changes in the BMEC permeability for HIV-1 and paracellular permeability are downstream of the MAPK signaling pathways. Pathways downstream of the cytokines are likely COX-2 for IL-6-induced changes in TEER [48] and the JAK/STAT pathway for IL-6 and GM-CSF [51,52] mediation of HIV-1 effects on immune cell migration [53]. Thus, IL-6 and GM-CSF likely increase HIV-1 transport across the BBB through other intracellular signaling pathways. As for the mechanisms by which LPS could increase HIV-1 transport across the BBB, the following sequential events are proposed: (1) LPS activates p44/42 MAPK and p38 MAPK in BMECs; (2) this activation induces BMECs to release IL-6 and GM-CSF into the blood; (3) IL-6 and GM-CSF act at the luminal surface of the BMECs to enhance the transcellular transport of HIV-1 across the BBB.

In our previous study, we demonstrated that p38 MAPK mediated LPS-enhanced HIV-1 transport and p44/42 MAPK mediated the LPS-induced increase in paracellular permeability using each pathway inhibitor [35]. U0126, the p44/42 MAPK inhibitor, did not attenuate LPS-enhanced HIV-1 transport. Here, U0126 as well as SB203580 decreased the release of IL-6 and GM-CSF (Figure 5). These findings suggest that the p38 MAPK signaling pathway directly leads to enhanced LPS-mediated transcellular transport of HIV-1.

In conclusion, we found that LPS potentiated the release of IL-6 and GM-CSF by BMECs through the activation of p44/42 MAPK and p38 MAPK. In addition to the p38 MAPK pathway, IL-6 and GM-CSF released from BECs acted at the luminal but not the abluminal surface to enhance HIV-1 transcellular transport. The p44/42 MAPK pathway and IL-6 likely acted at an intracellular site to increase paracellular permeability. Thus, LPS effects on HIV-permeation and on paracellular permeability were mediated through different cellular pathways. These results suggest that the release of cytokines by BECs plays an important role in the invasion of HIV-1 into the central nervous system. Preventing cytokine release by BECs through MAPK signaling pathways may be a therapeutic target in HIV-associated neurological dysfunction.

## Abbreviations

ANOVA: Analysis of variance; BBB: Blood-brain barrier; BEC: Brain endothelial cells; BMEC: Brain microvascular endothelial cells; cART: Combination antiretroviral therapy; GM-CSF: Granulocyte-macrophage colony-stimulating factor; HAART: Highly active antiretroviral therapy; HAD: HIV-1-associated dementia; HIV-1: Human immunodeficiency virus type 1; IL: Interleukin; IFN: Interferon; JNK: Jun kinase; LPS: Lipopolysaccharide; MAPK: Mitogen-activated protein kinase; MEK: MAPK kinase; *P*_e_: Permeability coefficient; PVDF: Polyvinylidene difluoride; TEER: Transendothelial electrical resistance; TGF: Transforming growth factor; TLR: Toll-like receptor; TNF-α: Tumor necrosis factor-α.

## Competing interests

The authors declare that they have no competing interests.

## Authors' contributions

All authors contributed to experimental design in an interactive and synergistic fashion. Experiments were performed by SD and MAF-D. Writing was a joint effort with WAB overseeing and editing final draft. All authors have read and validated the final manuscript.
